# Supplementary figures and images for: SynToxProfiler: An interactive analysis of drug combination synergy, toxicity and efficacy
Source: PLoS Comput Biol. 2020 Feb 3;16(2):e1007604. doi: 10.1371/journal.pcbi.1007604 (PMC7018095; doi:10.1371/journal.pcbi.1007604)

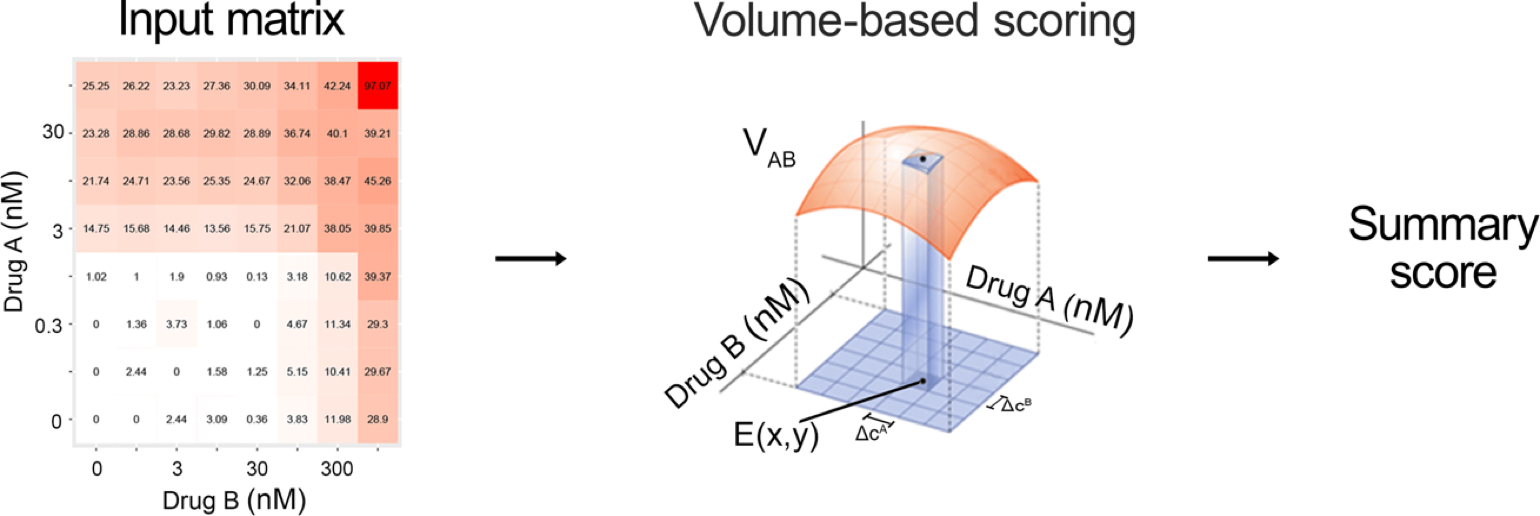

Supplement: S1 Fig — A schematic representation of calculation of combination efficacy, synergy and toxicity based on dose–response measurements on diseased cells or control cells. E(x,y) is the response at concentrations x and y of drugs A and B, respectively; ΔcA and ΔcB are the logarithmic increase in concentration of drug A and drug B between two consecutive measurements of the dose-response matrix. (TIF) [file pcbi.1007604.s001.tif]

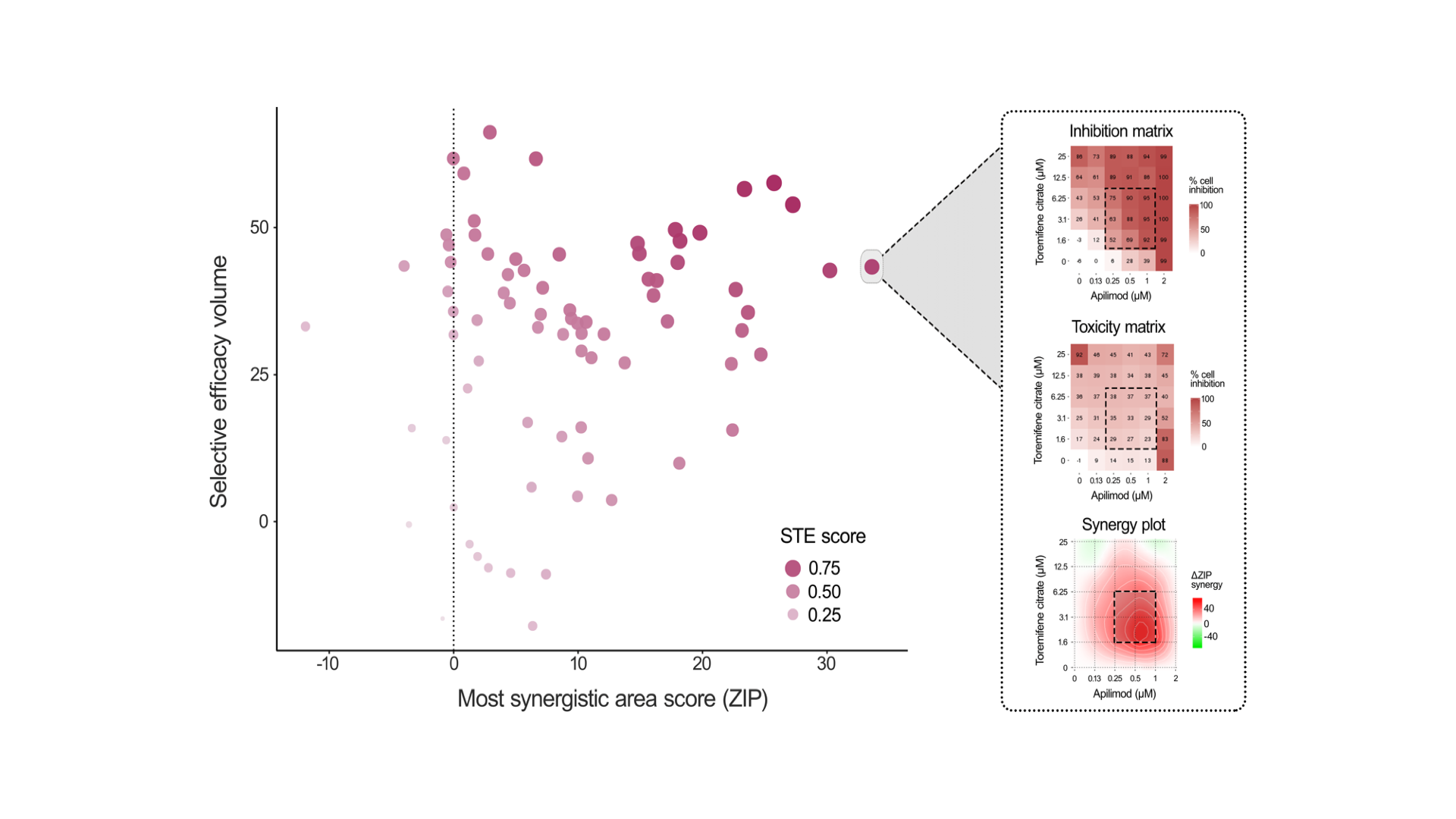

Supplement: S2 Fig — Scatter plot showing the distribution of a synergy score (x-axis) and selected efficacy score (y-axis) for 77 combinations tested in the Ebola infected and non–virus-infected Huh7 liver cells. Each drug combination is colored according to its STE score. Users can hover over the combinations to visualize their individual scores (e.g. STE score, or combination synergy, efficacy and toxicity scores), along with different dose-response matrices (synergy, toxicity, and efficacy), separately for each drug combination as shown here for the apilimod- toremifene citrate combination (right panel). (TIF) [file pcbi.1007604.s002.tif]

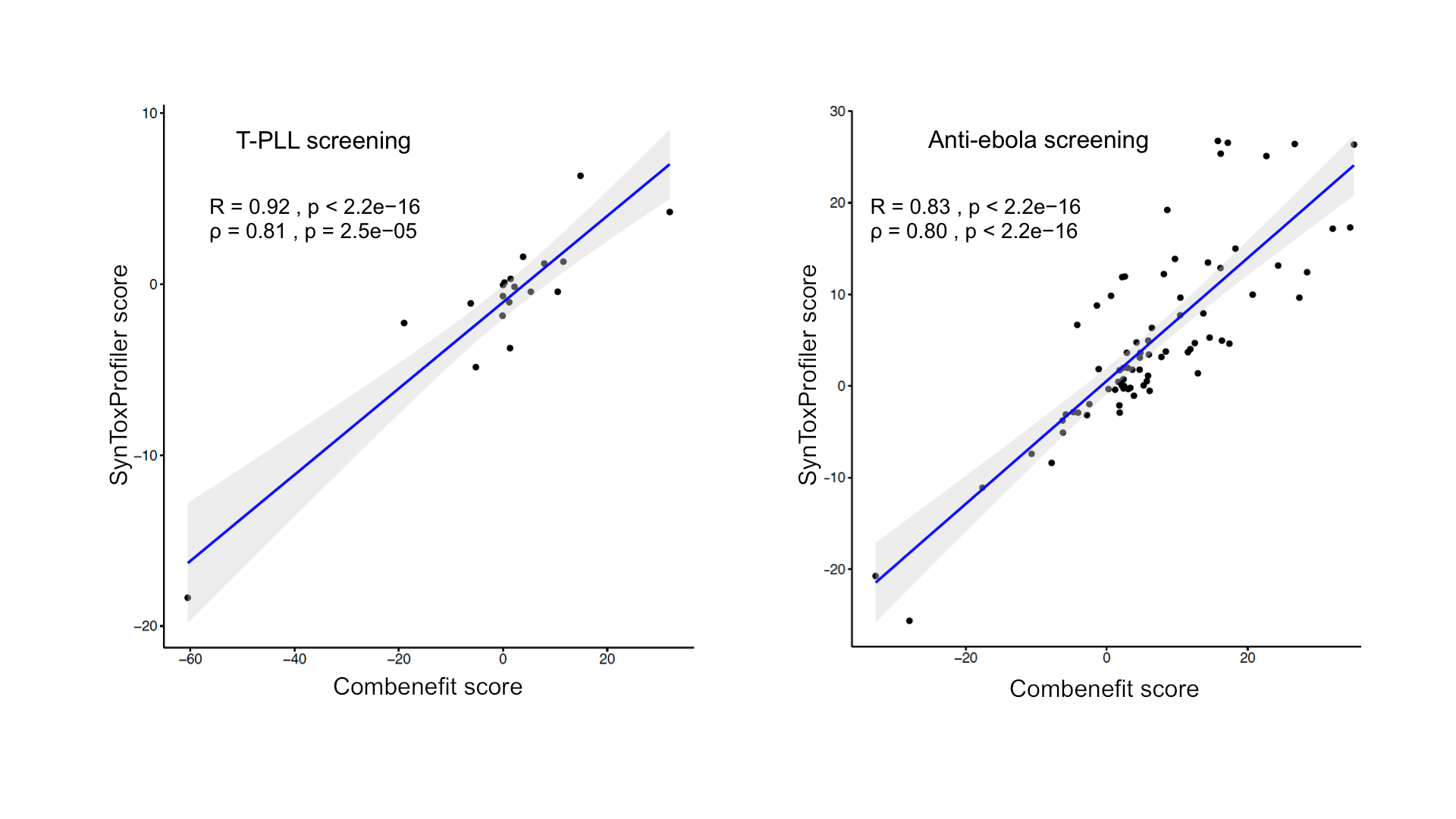

Supplement: S3 Fig — The pearson (R) and Spearman (ρ) correlation coefficients for each data along with respective correlation p-values are shown for both screens. The grey shaded area represents the 95% confidence interval for the fitted regression lines. For calculation of Combenefit synergy score, we have used the SUM_SYN_ANT score. (TIF) [file pcbi.1007604.s003.tif]

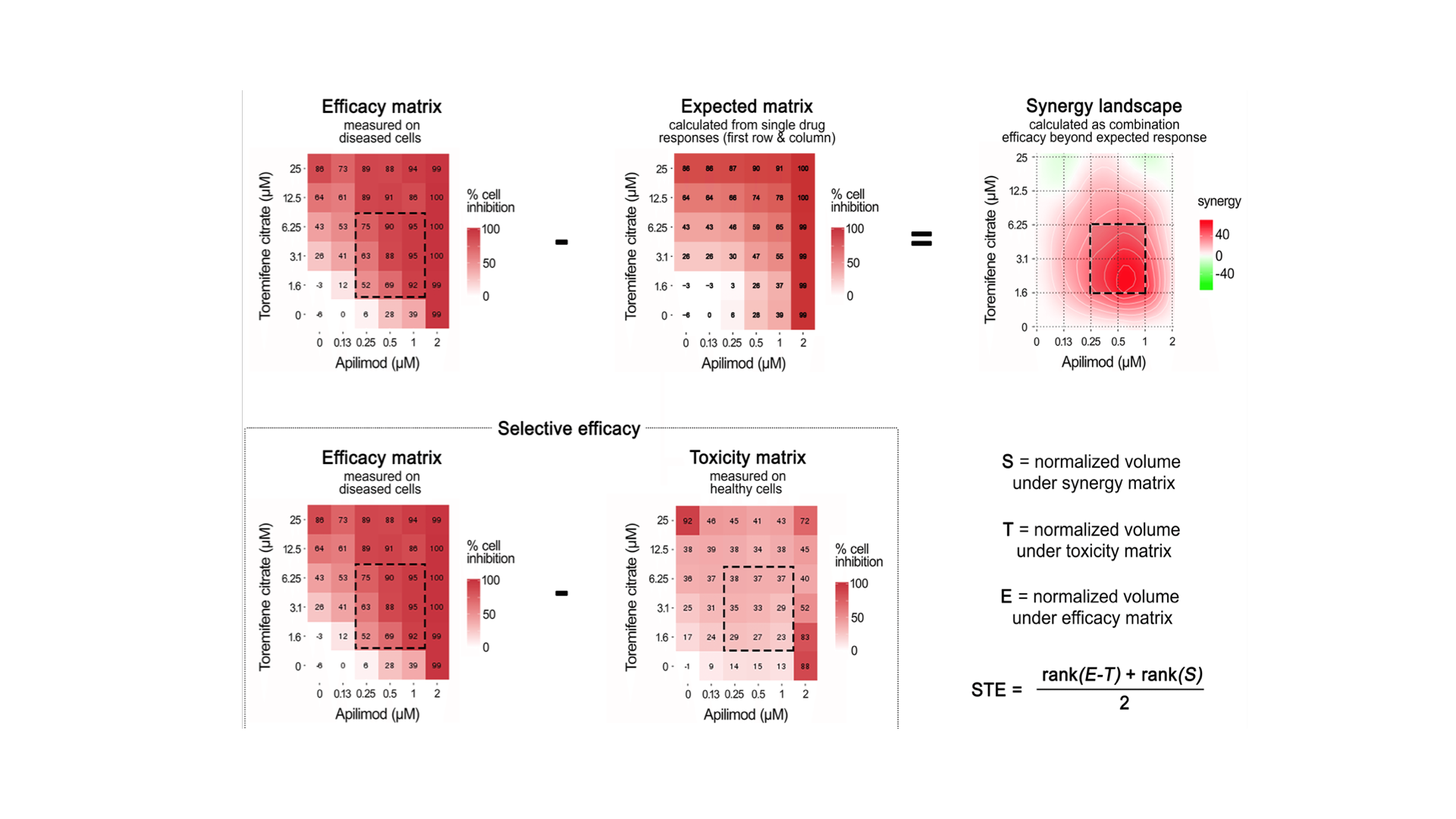

Supplement: S4 Fig — The user can choose whether the scores are calculated over the full dose-combination matrix, or over the most synergistic 3x3 dose window (the dotted square). (TIF) [file pcbi.1007604.s004.tif]
