# Supplementary material for: SynToxProfiler: An interactive analysis of drug combination synergy, toxicity and efficacy
Source: PLoS Comput Biol. 2020 Feb 3;16(2):e1007604. doi: 10.1371/journal.pcbi.1007604 (PMC7018095; doi:10.1371/journal.pcbi.1007604)
Supplement: S1 Table — (DOCX) [file pcbi.1007604.s005.docx]

| **Drug name** | **Targets** | **Mechanism** | **Approval status** |
| --- | --- | --- | --- |
| Ipatasertib | AKT inhibitor | Kinase inhibitor | Investigational |
| Daunorubicin | Topoisomerase II inhibitor | Chemotherapy | Approved |
| Doxorubicin | Topoisomerase II inhibitor | Chemotherapy | Approved |
| Omacetaxine | 80 S ribosome inhibitor | Chemotherapy | Approved |
| Navitoclax | Bcl-2/Bcl-xL inhibitor | Apoptotic modulator | Investigational |
| Idarubicin | Topoisomerase II inhibitor | Chemotherapy | Approved |
| S-63845 | MCL-1 inhibitor | Apoptotic modulator | Probe |
| Alpelisib | PI3Kalpha selective inhibitor | Kinase inhibitor | Investigational |
| Trametinib | MEK1/2 inhibitor | Kinase inhibitor | Approved |
| ASP3026 | ALK inhibitor | Kinase inhibitor | Investigational |
| Prexasertib | Chk1 inhibitor | Kinase inhibitor | Investigational |
| Dexamethasone | Glucocorticoid, immunomodulatory agent | Immunomodulatory | Approved |
| Clofarabine | Antimetabolite; Purine analog | Chemotherapy | Approved |
| Ibrutinib | Btk inhibitor | Kinase inhibitor | Approved |
| Dasatinib | Abl, Src, Kit, EphR Inhibitor | Kinase inhibitor | Approved |
| Cytarabine | Antimetabolite, interferes with DNA synthesis | Chemotherapy | Approved |
| Gefitinib | EGFR inhibitor | Kinase inhibitor | Approved |
| Carboplatin | Platinum-based antineoplastic agent | Chemotherapy | Approved |
| Vinorelbine | Mitotic inhibitor | Chemotherapy | Approved |
| Buparlisib | PI3K inhibitor | Kinase inhibitor | Investigational |
| Quizartinib | FLT3 inhibitor | Kinase inhibitor | Investigational |
|  |  |  |  |
