# Supplementary material for: SynToxProfiler: An interactive analysis of drug combination synergy, toxicity and efficacy
Source: PLoS Comput Biol. 2020 Feb 3;16(2):e1007604. doi: 10.1371/journal.pcbi.1007604 (PMC7018095; doi:10.1371/journal.pcbi.1007604)
Supplement: S2 Table — The STE score and respective ranks has been calculated for most synergistic area in each combination under ZIP synergy model. (DOCX) [file pcbi.1007604.s006.docx]

| **Drug1** | **Drug2** | **STE (ZIP)** | **Rank (ZIP)** | **STE (HSA)** | **Rank (HSA)** | **STE (Bliss)** | **Rank**  **(Bliss)** |
| --- | --- | --- | --- | --- | --- | --- | --- |
| Cytarabine | Daunorubicin | 0.83 | 1 | 0.85 | 1 | 0.83 | 1 |
| Trametinib | S-63845 | 0.83 | 2 | 0.78 | 3 | 0.8 | 2 |
| Ibrutinib | Navitoclax | 0.83 | 3 | 0.6 | 10 | 0.68 | 9 |
| Quizartinib | S-63845 | 0.75 | 4 | 0.7 | 5 | 0.7 | 4 |
| Omacetaxine | Ipatasertib | 0.73 | 5 | 0.63 | 9 | 0.68 | 8 |
| Gefitinib | Omacetaxine | 0.73 | 6 | 0.65 | 7 | 0.68 | 6 |
| Clofarabine | Idarubicin | 0.73 | 7 | 0.78 | 4 | 0.73 | 3 |
| Omacetaxine | Alpelisib | 0.68 | 8 | 0.6 | 11 | 0.65 | 10 |
| Clofarabine | Prexasertib | 0.68 | 9 | 0.65 | 8 | 0.68 | 7 |
| Gefitinib | Trametinib | 0.55 | 10 | 0.68 | 6 | 0.68 | 5 |
| Buparlisib | Ibrutinib | 0.55 | 11 | 0.55 | 12 | 0.58 | 11 |
| Ibrutinib | Doxorubicin | 0.53 | 12 | 0.45 | 14 | 0.48 | 13 |
| Vinorelbine | Clofarabine | 0.5 | 13 | 0.8 | 2 | 0.4 | 15 |
| Clofarabine | Omacetaxine | 0.38 | 14 | 0.31 | 16 | 0.38 | 16 |
| Dexamethasone | Clofarabine | 0.35 | 15 | 0.15 | 18 | 0.14 | 20 |
| Dasatinib | Ipatasertib | 0.25 | 16 | 0.43 | 15 | 0.45 | 14 |
| Carboplatin | Dexamethasone | 0.2 | 17 | 0.15 | 19 | 0.16 | 18 |
| Ipatasertib | ASP3026 | 0.18 | 18 | 0.11 | 20 | 0.15 | 19 |
| Idarubicin | Ibrutinib | 0.18 | 19 | 0.18 | 17 | 0.2 | 17 |
| Trametinib | Dasatinib | 0.1 | 20 | 0.48 | 13 | 0.5 | 12 |
