# Supplementary material for: SynToxProfiler: An interactive analysis of drug combination synergy, toxicity and efficacy
Source: PLoS Comput Biol. 2020 Feb 3;16(2):e1007604. doi: 10.1371/journal.pcbi.1007604 (PMC7018095; doi:10.1371/journal.pcbi.1007604)
Supplement: S3 Table — The STE score and respective ranks has been calculated for most synergistic area in each combination under ZIP synergy model. (DOCX) [file pcbi.1007604.s007.docx]

| **Drug1** | **Drug2** | **STE (ZIP)** | **Rank (ZIP)** | **STE (HSA)** | **Rank (HSA)** | **STE (Bliss)** | **Rank**  **(Bliss)** |
| --- | --- | --- | --- | --- | --- | --- | --- |
| Clomifene citrate | Sertraline hydrochloride | 0.96 | 1 | 0.93 | 2 | 0.94 | 1 |
| Sertraline hydrochloride | Toremifene citrate | 0.95 | 2 | 0.94 | 1 | 0.93 | 3 |
| Clomifene citrate | Colchicine | 0.93 | 3 | 0.88 | 5 | 0.88 | 4 |
| Colchicine | 3-Deazaneplanocin A | 0.87 | 4 | 0.84 | 7 | 0.86 | 5 |
| Toremifene citrate | Apilimod | 0.86 | 5 | 0.83 | 10 | 0.83 | 6 |
| Colchicine | Toremifene citrate | 0.85 | 6 | 0.85 | 6 | 0.81 | 9 |
| Sertraline hydrochloride | Sertraline hydrochloride | 0.84 | 7 | 0.82 | 11 | 0.75 | 12 |
| Clomifene citrate | Apilimod | 0.84 | 8 | 0.90 | 4 | 0.94 | 2 |
| Sertraline hydrochloride | 3-Deazaneplanocin A | 0.78 | 9 | 0.84 | 8 | 0.76 | 11 |
| Toremifene citrate | 3-Deazaneplanocin A | 0.78 | 10 | 0.93 | 3 | 0.83 | 7 |
| Colchicine | Mycophenolate mofetil | 0.77 | 11 | 0.72 | 19 | 0.68 | 19 |
| Sunitinib malate | Toremifene citrate | 0.76 | 12 | 0.72 | 18 | 0.71 | 15 |
| Colchicine | Aripiprazole | 0.73 | 13 | 0.42 | 49 | 0.75 | 13 |
| Mycophenolate mofetil | 3-Deazaneplanocin A | 0.71 | 14 | 0.73 | 16 | 0.65 | 21 |
| Omacetaxine mepesuccinate | Toremifene citrate | 0.71 | 15 | 0.43 | 46 | 0.69 | 18 |
| 3-Deazaneplanocin A | Favipiravir | 0.71 | 16 | 0.42 | 50 | 0.59 | 30 |
| 3-Deazaneplanocin A | Apilimod | 0.68 | 17 | 0.84 | 9 | 0.83 | 8 |
| Omacetaxine mepesuccinate | Piperacetazine | 0.66 | 18 | 0.60 | 24 | 0.66 | 20 |
| Colchicine | Sertraline hydrochloride | 0.66 | 19 | 0.78 | 13 | 0.78 | 10 |
| Clomifene citrate | Toremifene citrate | 0.65 | 20 | 0.73 | 15 | 0.71 | 16 |
| Apilimod | Piperacetazine | 0.64 | 21 | 0.61 | 23 | 0.60 | 26 |
| Colchicine | Sunitinib malate | 0.64 | 22 | 0.71 | 20 | 0.62 | 23 |
| Mycophenolate mofetil | Sertraline hydrochloride | 0.58 | 23 | 0.75 | 14 | 0.60 | 27 |
| Sertraline hydrochloride | Sunitinib malate | 0.58 | 24 | 0.51 | 37 | 0.55 | 37 |
| Toremifene citrate | Toremifene citrate | 0.58 | 25 | 0.59 | 26 | 0.57 | 34 |
| Omacetaxine mepesuccinate | Omacetaxine mepesuccinate | 0.57 | 26 | 0.54 | 32 | 0.58 | 32 |
| Omacetaxine mepesuccinate | 3-Deazaneplanocin A | 0.57 | 27 | 0.55 | 30 | 0.58 | 33 |
| Apilimod | Apilimod | 0.57 | 28 | 0.59 | 27 | 0.54 | 38 |
| 3-Deazaneplanocin A | Piperacetazine | 0.56 | 29 | 0.48 | 39 | 0.60 | 28 |
| Colchicine | Favipiravir | 0.56 | 30 | 0.32 | 59 | 0.57 | 35 |
| Omacetaxine mepesuccinate | Sunitinib malate | 0.55 | 31 | 0.44 | 44 | 0.53 | 39 |
| Colchicine | Piperacetazine | 0.55 | 32 | 0.50 | 38 | 0.60 | 29 |
| Clomifene citrate | Sunitinib malate | 0.55 | 33 | 0.64 | 22 | 0.64 | 22 |
| Omacetaxine mepesuccinate | Sertraline hydrochloride | 0.55 | 34 | 0.48 | 40 | 0.53 | 42 |
| Sunitinib malate | 3-Deazaneplanocin A | 0.54 | 35 | 0.57 | 29 | 0.53 | 40 |
| Clomifene citrate | Mycophenolate mofetil | 0.54 | 36 | 0.73 | 17 | 0.53 | 41 |
| Toremifene citrate | Piperacetazine | 0.53 | 37 | 0.51 | 35 | 0.43 | 48 |
| Mycophenolate mofetil | Toremifene citrate | 0.51 | 38 | 0.67 | 21 | 0.59 | 31 |
| 3-Deazaneplanocin A | Aripiprazole | 0.51 | 39 | 0.21 | 71 | 0.43 | 49 |
| Sertraline hydrochloride | Piperacetazine | 0.51 | 40 | 0.52 | 33 | 0.60 | 25 |
| Sunitinib malate | Sunitinib malate | 0.50 | 41 | 0.57 | 28 | 0.57 | 36 |
| Clomifene citrate | Piperacetazine | 0.50 | 42 | 0.25 | 70 | 0.16 | 71 |
| Toremifene citrate | Favipiravir | 0.50 | 43 | 0.31 | 62 | 0.44 | 47 |
| Clomifene citrate | 3-Deazaneplanocin A | 0.49 | 44 | 0.55 | 31 | 0.62 | 24 |
| Omacetaxine mepesuccinate | Favipiravir | 0.48 | 45 | 0.42 | 51 | 0.45 | 46 |
| Sunitinib malate | Aripiprazole | 0.47 | 46 | 0.45 | 43 | 0.46 | 45 |
| Clomifene citrate | Omacetaxine mepesuccinate | 0.47 | 47 | 0.40 | 54 | 0.42 | 51 |
| Mycophenolate mofetil | Sunitinib malate | 0.47 | 48 | 0.80 | 12 | 0.70 | 17 |
| Mycophenolate mofetil | Piperacetazine | 0.46 | 49 | 0.40 | 53 | 0.29 | 62 |
| Sunitinib malate | Apilimod | 0.45 | 50 | 0.43 | 47 | 0.42 | 52 |
| Omacetaxine mepesuccinate | Apilimod | 0.44 | 51 | 0.44 | 45 | 0.38 | 54 |
| Colchicine | Apilimod | 0.43 | 52 | 0.47 | 41 | 0.47 | 44 |
| Piperacetazine | Piperacetazine | 0.42 | 53 | 0.33 | 58 | 0.22 | 65 |
| Sunitinib malate | Piperacetazine | 0.42 | 54 | 0.34 | 56 | 0.37 | 55 |
| Clomifene citrate | Clomifene citrate | 0.38 | 55 | 0.52 | 34 | 0.43 | 50 |
| Sunitinib malate | Favipiravir | 0.37 | 56 | 0.28 | 64 | 0.36 | 56 |
| Mycophenolate mofetil | Favipiravir | 0.37 | 57 | 0.59 | 25 | 0.71 | 14 |
| Clomifene citrate | Favipiravir | 0.36 | 58 | 0.33 | 57 | 0.31 | 60 |
| Mycophenolate mofetil | Mycophenolate mofetil | 0.36 | 59 | 0.51 | 36 | 0.31 | 59 |
| Omacetaxine mepesuccinate | Aripiprazole | 0.34 | 60 | 0.27 | 65 | 0.34 | 58 |
| Sertraline hydrochloride | Aripiprazole | 0.34 | 61 | 0.32 | 60 | 0.30 | 61 |
| Mycophenolate mofetil | Apilimod | 0.30 | 62 | 0.43 | 48 | 0.27 | 63 |
| Piperacetazine | Favipiravir | 0.29 | 63 | 0.25 | 68 | 0.21 | 66 |
| Sertraline hydrochloride | Apilimod | 0.27 | 64 | 0.31 | 63 | 0.27 | 64 |
| Colchicine | Omacetaxine mepesuccinate | 0.27 | 65 | 0.46 | 42 | 0.50 | 43 |
| Mycophenolate mofetil | Aripiprazole | 0.24 | 66 | 0.25 | 69 | 0.21 | 67 |
| Clomifene citrate | Aripiprazole | 0.23 | 67 | 0.25 | 67 | 0.42 | 53 |
| 3-Deazaneplanocin A | 3-Deazaneplanocin A | 0.23 | 68 | 0.31 | 61 | 0.20 | 70 |
| Piperacetazine | Aripiprazole | 0.21 | 69 | 0.26 | 66 | 0.21 | 68 |
| Aripiprazole | Aripiprazole | 0.19 | 70 | 0.12 | 75 | 0.20 | 69 |
| Sertraline hydrochloride | Favipiravir | 0.18 | 71 | 0.05 | 77 | 0.08 | 76 |
| Favipiravir | Favipiravir | 0.16 | 72 | 0.16 | 73 | 0.14 | 73 |
| Mycophenolate mofetil | Omacetaxine mepesuccinate | 0.15 | 73 | 0.09 | 76 | 0.14 | 74 |
| Toremifene citrate | Aripiprazole | 0.14 | 74 | 0.17 | 72 | 0.09 | 75 |
| Apilimod | Aripiprazole | 0.14 | 75 | 0.35 | 55 | 0.16 | 72 |
| Aripiprazole | Favipiravir | 0.07 | 76 | 0.13 | 74 | 0.07 | 77 |
| Apilimod | Favipiravir | 0.05 | 77 | 0.41 | 52 | 0.34 | 57 |
