# Supplementary material for: SynToxProfiler: An interactive analysis of drug combination synergy, toxicity and efficacy
Source: PLoS Comput Biol. 2020 Feb 3;16(2):e1007604. doi: 10.1371/journal.pcbi.1007604 (PMC7018095; doi:10.1371/journal.pcbi.1007604)
Supplement: S4 Table — The rank of Bliss synergy and STE scores calculated for full synergy matrix by SynToxProfiler have been compared against SUM_SYN_ANT synergy score from Combenefit. (DOCX) [file pcbi.1007604.s008.docx]

| Combination | STE_SynToxProfiler_ | Rank  (STE_SytnToxProfiler_) | Synergy  _SynToxprofiler_ | Rank  (Synergy_SynToxprofiler_) | Synergy  _Combenefit_ | Rank  (Synergy_combenefit_) |
| --- | --- | --- | --- | --- | --- | --- |
| Cytarabine-Daunorubicin | 0.83 | 1 | 6.33 | 2 | 14.9 | 2 |
| Trametinib-S-63845 | 0.8 | 2 | 4.25 | 1 | 32.0 | 1 |
| Clofarabine-Idarubicin | 0.73 | 3 | 1.32 | 8 | 11.6 | 3 |
| Quizartinib-S-63845 | 0.7 | 4 | -1.05 | 4 | 1.2 | 11 |
| Gefitinib-Trametinib | 0.68 | 5 | -0.44 | 3 | 10.5 | 4 |
| Clofarabine-Prexasertib | 0.68 | 6 | 1.24 | 7 | 7.9 | 5 |
| Ibrutinib-Navitoclax | 0.68 | 7 | 1.61 | 10 | 3.8 | 7 |
| Gefitinib-Omacetaxine | 0.68 | 8 | 0.33 | 11 | 1.5 | 9 |
| Omacetaxine-Ipatasertib | 0.68 | 9 | 0.11 | 12 | 0.3 | 12 |
| Omacetaxine-Alpelisib | 0.65 | 10 | -1.11 | 14 | -6.2 | 17 |
| Buparlisib-Ibrutinib | 0.58 | 11 | -0.44 | 5 | 5.3 | 6 |
| Trametinib-Dasatinib | 0.5 | 12 | -3.73 | 6 | 1.3 | 10 |
| Dasatinib-Ipatasertib | 0.45 | 13 | -0.13 | 9 | 2.2 | 8 |
| Vinorelbine-Clofarabine | 0.4 | 14 | -2.27 | 19 | -19.0 | 18 |
| Clofarabine-Omacetaxine | 0.38 | 15 | -4.85 | 15 | -5.2 | 16 |
| Idarubicin-Ibrutinib | 0.2 | 16 | -1.83 | 13 | -0.1 | 15 |
| Carboplatin-Dexamethasone | 0.16 | 17 | -0.69 | 17 | 0.0 | 14 |
| Ipatasertib-ASP3026 | 0.15 | 18 | -0.02 | 16 | 0.0 | 13 |
| Dexamethasone-Clofarabine | 0.14 | 19 | -18.34 | 18 | -60.5 | 19 |
