# Supplementary material for: SynToxProfiler: An interactive analysis of drug combination synergy, toxicity and efficacy
Source: PLoS Comput Biol. 2020 Feb 3;16(2):e1007604. doi: 10.1371/journal.pcbi.1007604 (PMC7018095; doi:10.1371/journal.pcbi.1007604)
Supplement: S5 Table — The rank of Bliss synergy and STE scores calculated for full synergy matrix by SynToxProfiler have been compared against SUM_SYN_ANT synergy score from Combenefit. (DOCX) [file pcbi.1007604.s009.docx]

| Combination | STE_SynToxProfiler_ | Rank  (STE_SytnToxProfiler_) | Synergy  _SynToxprofiler_ | Rank  (Synergy_SynToxprofiler_) | Synergy  _Combenefit_ | Rank  (Synergy_combenefit_) |
| --- | --- | --- | --- | --- | --- | --- |
| Clomifenecitrate_Colchicine | 0.94 | 1 | 25.12 | 6 | 22.66 | 8 |
| Colchicine_3-DeazaneplanocinA | 0.93 | 2 | 26.79 | 1 | 15.79 | 16 |
| Clomifenecitrate_Apilimod | 0.92 | 3 | 26.41 | 4 | 35.08 | 1 |
| Colchicine_Toremifenecitrate | 0.92 | 4 | 13.51 | 12 | 14.38 | 18 |
| Toremifenecitrate_3-DeazaneplanocinA | 0.90 | 5 | 19.21 | 7 | 8.59 | 27 |
| Colchicine_Sertralinehydrochloride | 0.86 | 6 | 26.46 | 3 | 26.68 | 6 |
| Sertralinehydrochloride_Toremifenecitrate | 0.83 | 7 | 9.67 | 21 | 27.33 | 5 |
| Toremifenecitrate_Apilimod | 0.81 | 8 | 17.22 | 9 | 32.07 | 3 |
| Sertralinehydrochloride_3-DeazaneplanocinA | 0.81 | 9 | 12.22 | 16 | 8.11 | 29 |
| 3-DeazaneplanocinA_Apilimod | 0.79 | 10 | 13.90 | 11 | 9.70 | 26 |
| Clomifenecitrate_Sunitinibmalate | 0.79 | 11 | 4.67 | 33 | 17.41 | 11 |
| Colchicine_Mycophenolatemofetil | 0.77 | 12 | 4.96 | 30 | 5.89 | 34 |
| Colchicine_Aripiprazole | 0.76 | 13 | 25.39 | 5 | 16.18 | 14 |
| Mycophenolatemofetil_Favipiravir | 0.76 | 14 | 3.63 | 38 | 2.86 | 47 |
| Sertralinehydrochloride_Sertralinehydrochloride | 0.75 | 15 | 12.44 | 15 | 28.43 | 4 |
| 3-DeazaneplanocinA_Favipiravir | 0.75 | 16 | 8.79 | 23 | -1.39 | 64 |
| Clomifenecitrate_Sertralinehydrochloride | 0.75 | 17 | 17.31 | 8 | 34.54 | 2 |
| Clomifenecitrate_Mycophenolatemofetil | 0.72 | 18 | 6.37 | 27 | 6.41 | 31 |
| Clomifenecitrate_3-DeazaneplanocinA | 0.70 | 19 | 14.99 | 10 | 18.28 | 10 |
| Colchicine_Sunitinibmalate | 0.68 | 20 | 12.91 | 14 | 16.15 | 15 |
| Mycophenolatemofetil_Sunitinibmalate | 0.68 | 21 | 2.02 | 42 | 2.36 | 53 |
| 3-DeazaneplanocinA_Piperacetazine | 0.68 | 22 | 9.88 | 20 | 0.62 | 61 |
| Mycophenolatemofetil_Sertralinehydrochloride | 0.68 | 23 | 1.41 | 48 | 12.93 | 20 |
| Colchicine_Piperacetazine | 0.67 | 24 | 26.59 | 2 | 17.17 | 12 |
| Colchicine_Favipiravir | 0.65 | 25 | 12.01 | 17 | 2.58 | 48 |
| 3-DeazaneplanocinA_Aripiprazole | 0.65 | 26 | 6.73 | 26 | -4.16 | 68 |
| Sunitinibmalate_Toremifenecitrate | 0.61 | 27 | 3.75 | 36 | 11.54 | 23 |
| Omacetaxinemepesuccinate_Piperacetazine | 0.59 | 28 | 0.76 | 51 | 2.38 | 51 |
| Colchicine_Apilimod | 0.56 | 29 | 11.89 | 18 | 2.20 | 54 |
| Mycophenolatemofetil_Piperacetazine | 0.55 | 30 | 1.83 | 45 | 3.64 | 43 |
| Toremifenecitrate_Piperacetazine | 0.54 | 31 | 3.64 | 37 | 4.77 | 38 |
| Omacetaxinemepesuccinate_Toremifenecitrate | 0.53 | 32 | 1.79 | 46 | 4.66 | 40 |
| Clomifenecitrate_Toremifenecitrate | 0.51 | 33 | 5.33 | 28 | 14.61 | 17 |
| Mycophenolatemofetil_Mycophenolatemofetil | 0.51 | 34 | -5.03 | 72 | -6.21 | 71 |
| Apilimod_Piperacetazine | 0.51 | 35 | 13.19 | 13 | 24.33 | 7 |
| Sertralinehydrochloride_Sunitinibmalate | 0.51 | 36 | 9.97 | 19 | 20.70 | 9 |
| Colchicine_Omacetaxinemepesuccinate | 0.50 | 37 | -1.02 | 63 | 3.88 | 42 |
| Mycophenolatemofetil_3-DeazaneplanocinA | 0.49 | 38 | 7.74 | 25 | 10.44 | 25 |
| Apilimod_Apilimod | 0.48 | 39 | 3.76 | 35 | 8.40 | 28 |
| Omacetaxinemepesuccinate_Favipiravir | 0.48 | 40 | -0.24 | 58 | 2.39 | 50 |
| Sunitinibmalate_3-DeazaneplanocinA | 0.47 | 41 | 9.64 | 22 | 10.47 | 24 |
| Clomifenecitrate_Clomifenecitrate | 0.47 | 42 | -3.17 | 70 | -2.79 | 66 |
| Mycophenolatemofetil_Aripiprazole | 0.46 | 43 | -1.94 | 64 | -2.45 | 65 |
| Toremifenecitrate_Toremifenecitrate | 0.46 | 44 | -2.82 | 66 | -4.72 | 69 |
| Sunitinibmalate_Favipiravir | 0.46 | 45 | 3.10 | 41 | 4.69 | 39 |
| Mycophenolatemofetil_Toremifenecitrate | 0.45 | 46 | 1.99 | 43 | 2.98 | 46 |
| Sertralinehydrochloride_Piperacetazine | 0.44 | 47 | 7.92 | 24 | 13.71 | 19 |
| Omacetaxinemepesuccinate_Apilimod | 0.44 | 48 | -0.31 | 60 | 3.07 | 45 |
| Omacetaxinemepesuccinate_Sunitinibmalate | 0.44 | 49 | 0.08 | 54 | 5.23 | 37 |
| Omacetaxinemepesuccinate_3-DeazaneplanocinA | 0.42 | 50 | 0.78 | 50 | 2.36 | 52 |
| Sertralinehydrochloride_Aripiprazole | 0.40 | 51 | 4.03 | 34 | 11.86 | 22 |
| Omacetaxinemepesuccinate_Sertralinehydrochloride | 0.39 | 52 | 0.46 | 53 | 1.64 | 59 |
| Sunitinibmalate_Sunitinibmalate | 0.38 | 53 | 3.19 | 40 | 7.77 | 30 |
| Toremifenecitrate_Favipiravir | 0.38 | 54 | 1.86 | 44 | -1.12 | 63 |
| Sunitinibmalate_Piperacetazine | 0.37 | 55 | 4.97 | 29 | 16.34 | 13 |
| Sunitinibmalate_Apilimod | 0.37 | 56 | 1.18 | 49 | 5.86 | 35 |
| Clomifenecitrate_Aripiprazole | 0.36 | 57 | 4.70 | 32 | 12.51 | 21 |
| Clomifenecitrate_Omacetaxinemepesuccinate | 0.36 | 58 | -0.38 | 61 | 1.19 | 60 |
| Omacetaxinemepesuccinate_Omacetaxinemepesuccinate | 0.34 | 59 | 0.02 | 56 | 2.46 | 49 |
| Mycophenolatemofetil_Apilimod | 0.33 | 60 | 1.72 | 47 | 1.82 | 57 |
| Piperacetazine_Aripiprazole | 0.33 | 61 | 4.77 | 31 | 4.24 | 41 |
| Sunitinibmalate_Aripiprazole | 0.32 | 62 | -2.07 | 65 | 1.79 | 58 |
| 3-DeazaneplanocinA_3-DeazaneplanocinA | 0.32 | 63 | -20.70 | 76 | -32.81 | 77 |
| Sertralinehydrochloride_Apilimod | 0.30 | 64 | 3.46 | 39 | 5.98 | 33 |
| Toremifenecitrate_Aripiprazole | 0.27 | 65 | -2.88 | 68 | 1.84 | 56 |
| Apilimod_Aripiprazole | 0.27 | 66 | -0.53 | 62 | 6.10 | 32 |
| Omacetaxinemepesuccinate_Aripiprazole | 0.21 | 67 | -0.19 | 57 | 3.36 | 44 |
| Favipiravir_Favipiravir | 0.21 | 68 | 0.53 | 52 | 5.71 | 36 |
| Mycophenolatemofetil_Omacetaxinemepesuccinate | 0.20 | 69 | -0.29 | 59 | 0.24 | 62 |
| Piperacetazine_Favipiravir | 0.18 | 70 | 0.08 | 55 | 2.16 | 55 |
| Clomifenecitrate_Piperacetazine | 0.18 | 71 | -7.39 | 73 | -10.66 | 74 |
| Apilimod_Favipiravir | 0.16 | 72 | -3.08 | 69 | -5.80 | 70 |
| Clomifenecitrate_Favipiravir | 0.14 | 73 | -3.74 | 71 | -6.26 | 72 |
| Sertralinehydrochloride_Favipiravir | 0.14 | 74 | -2.85 | 67 | -4.07 | 67 |
| Piperacetazine_Piperacetazine | 0.05 | 75 | -8.37 | 74 | -7.82 | 73 |
| Aripiprazole_Aripiprazole | 0.03 | 76 | -11.04 | 75 | -17.64 | 75 |
| Aripiprazole_Favipiravir | 0.01 | 77 | -25.57 | 77 | -28.00 | 76 |
