# Supplementary material for: SynToxProfiler: An interactive analysis of drug combination synergy, toxicity and efficacy
Source: PLoS Comput Biol. 2020 Feb 3;16(2):e1007604. doi: 10.1371/journal.pcbi.1007604 (PMC7018095; doi:10.1371/journal.pcbi.1007604)
Supplement: S1 Text — (DOCX) [file pcbi.1007604.s012.docx]

**Supplementary information for**

**SynToxProfiler: interactive analysis of drug combination synergy, toxicity and efficacy**

Aleksandr Ianevski^1,2^_,_ Sanna Timonen^1^, Alexander Kononov^1^, Tero Aittokallio^1,2,3*^, Anil K Giri^1*^

^1^ Institute for Molecular Medicine Finland (FIMM), University of Helsinki, FI-00290 Helsinki, Finland

^2^ Helsinki Institute for Information Technology (HIIT), Aalto University, FI-02150 Espoo, Finland

^3^ Department of Mathematics and Statistics, University of Turku, Quantum, FI-20014 Turku, Finland

^*^Corresponding authors

*Calculation of normalized volume score for higher-order combinations*

For the case of higher-order combinations (combination of 3 or more drugs), the normalized volume under the multidimensional dose-response surface is calculated while quantifying combination efficacy and toxicity based on measurements on diseased and control cells, respectively. Synergy score is estimated based on measurements on diseased cells and expected combination responses are determined by one of the synergy models applicable to higher-order combinations (e.g. Bliss or HSA). For example, for three-drug combination ABD of drugs A, B and D, the efficacy score (E_ABD_) is calculated as volume under the multidimensional dose-response surface given by:

$$E_{\mathrm{ABD}}=\frac{\sum_{x=C_{\min}^{A}}^{C_{\max}^{A}} \sum_{y=C_{\min}^{B}}^{C_{\max}^{B}} \sum_{z=C_{\min}^{D}}^{C_{\max}^{D}} E(x,y,z)\Delta c^{A}\Delta c^{B}\Delta c^{D}}{\ln\left( C_{\max}^{A}/C_{\min}^{A} \right) ln(C_{\max}^{B}/C_{\min}^{B})ln(C_{\max}^{D}/C_{\min}^{D})} . Eq. (5)$$

Here, c^A^_min_ and c^A^_max_ are the minimum and maximum tested concentrations of drug A, respectively, and c^B^_min_, and c^B^_max_ are those of drug B and c^D^_min_, and c^D^_max_ are those of drug D; Δc^A^, Δc^B^ and Δc^D^ are the logarithmic increase in concentration of drugs A, B and D between two consecutive measurements of multidimensional dose-response matrix; and E(x, y, z), is the efficacy toxicity or synergy levels at concentration x of drug A, concentration y of drug B and concentration z of drug D respectively.
