# Supplementary material for: SynToxProfiler: An interactive analysis of drug combination synergy, toxicity and efficacy
Source: PLoS Comput Biol. 2020 Feb 3;16(2):e1007604. doi: 10.1371/journal.pcbi.1007604 (PMC7018095; doi:10.1371/journal.pcbi.1007604)
Supplement: Attachment — Submitted filename: Responses_to_reviewers.docx [file pcbi.1007604.s013.docx]

**Comments to the Authors:**

***Reviewer #1:*** *Comments: A web-based tool is presented that allows the calculation of drug combination synergy, toxicity and efficacy. The justification for the investigation is that most methods that evaluate drug combinations do so based on a synergy parameter, such as the Bliss independence value, rather than an integrated metric that combines drug toxicity (need control cell data) and efficacy and synergy. Overall, this is a nice addition to the drug combination screening literature; however, there are a number of points that need to be addressed.*

**Our response:** We thank the reviewer for appreciating the added value of SynToxProfiler tool for drug combination screening. Please find below our point-by-point responses to the comments. We have highlighted in yellow all the changes made in the revised manuscript.

*It is hard to follow all the mathematical steps to attain an STE. They need to show all the equations for EAB, TAB and STEAB and use real numbers. Equation 1 appears to be the basis of the calculations, but a step-by-step outline is required. Further, how does the choice of synergistic model effect the STE calculation?*

**Our response:** We have now modified the method section, as suggested by the reviewer, and provided separate equations for the calculation of the efficacy score (E_AB_), toxicity score (T_AB_), and synergy score (S_AB_), as well as the combined synergy, efficacy and toxicity score (STE_AB_) as equations 1, 2, 3 and 4, respectively (please see, page 8-9 in the methods section).

To better illustrate the step-by-step scoring procedure with real input data, we have also added the below schematic illustration both as new S4 Fig and into the SynToxProfiler User Documentation (<https://syntoxprofiler.fimm.fi/howto>). In this example, the expected matrix is calculated using Bliss synergy model, but we have also tested other synergy models (see below).

For those users interested in seeing the actual calculations in the implementation, and to guarantee the replicability of the work and possibilities for further extension of the tool by academic communities, we have also provided the source code of the method at <https://github.com/IanevskiAleksandr/SynToxProfiler>.

As further suggested by the reviewer, we have now compared the STE score when calculated either using Zero Interaction Potency (ZIP), Bliss, or Highest Single Agent (HSA) models, for the 20 drug combinations tested in-house in T-PLL patient. We found that 9 of the top 10 combinations identified based on the STE score using ZIP model are actually common across all the 3 synergy models (see table below, new S2 Table and S3 Table). These results indicate that the choice of the synergy model does not have a significant effect on the overall ranks based on combined STE score (page 5, middle paragraph). However, we recommend that the users should choose the appropriate synergy model based on their underlying hypothesis behind drug interaction, as the different synergy models have different assumptions for the synergy calculation (page 7, first paragraph). For example, one should use Bliss synergy when the assumption is that drugs in combination act independently through different mechanisms, but together they can exceed their individual responses.

| **Drug1** | **Drug2** | **STE (ZIP)** | **Rank (ZIP)** | **STE (HSA)** | **Rank (HSA)** | **STE (Bliss)** | **Rank**  **(Bliss)** |
| --- | --- | --- | --- | --- | --- | --- | --- |
| Cytarabine | Daunorubicin | 0.83 | 1 | 0.85 | 1 | 0.83 | 1 |
| Trametinib | S-63845 | 0.83 | 2 | 0.78 | 3 | 0.8 | 2 |
| Ibrutinib | Navitoclax | 0.83 | 3 | 0.6 | 10 | 0.68 | 9 |
| Quizartinib | S-63845 | 0.75 | 4 | 0.7 | 5 | 0.7 | 4 |
| Omacetaxine | Ipatasertib | 0.73 | 5 | 0.63 | 9 | 0.68 | 8 |
| Gefitinib | Omacetaxine | 0.73 | 6 | 0.65 | 7 | 0.68 | 6 |
| Clofarabine | Idarubicin | 0.73 | 7 | 0.78 | 4 | 0.73 | 3 |
| Omacetaxine | Alpelisib | 0.68 | 8 | 0.6 | 11 | 0.65 | 10 |
| Clofarabine | Prexasertib | 0.68 | 9 | 0.65 | 8 | 0.68 | 7 |
| Gefitinib | Trametinib | 0.55 | 10 | 0.68 | 6 | 0.68 | 5 |
| Buparlisib | Ibrutinib | 0.55 | 11 | 0.55 | 12 | 0.58 | 11 |
| Ibrutinib | Doxorubicin | 0.53 | 12 | 0.45 | 14 | 0.48 | 13 |
| Vinorelbine | Clofarabine | 0.5 | 13 | 0.8 | 2 | 0.4 | 15 |
| Clofarabine | Omacetaxine | 0.38 | 14 | 0.31 | 16 | 0.38 | 16 |
| Dexamethasone | Clofarabine | 0.35 | 15 | 0.15 | 18 | 0.14 | 20 |
| Dasatinib | Ipatasertib | 0.25 | 16 | 0.43 | 15 | 0.45 | 14 |
| Carboplatin | Dexamethasone | 0.2 | 17 | 0.15 | 19 | 0.16 | 18 |
| Ipatasertib | ASP3026 | 0.18 | 18 | 0.11 | 20 | 0.15 | 19 |
| Idarubicin | Ibrutinib | 0.18 | 19 | 0.18 | 17 | 0.2 | 17 |
| Trametinib | Dasatinib | 0.1 | 20 | 0.48 | 13 | 0.5 | 12 |

We have chosen to use the ZIP model as the default option in the SynToxProfiler tool due to its beneficial and robust properties [see ref. 8], and therefore the example results in manuscript are based on STE score calculated based on ZIP model. However, we have added the comparison results across the synergy models as S2 Table (for T-PLL screening) and S3 Table (anti-Ebola screening), and briefly cited them in the results section (page 5, middle paragraph).

*- The website is quite reasonable, but it could be improved with more detailed directions. For example, step 1 might be how to create the excel file and comment on the need to specify units and format. One can find this information on the website but I feel it could be more clearly presented.*

**Our response:** We thank the reviewer for the suggestion. We have now added a *“How to use”* section to our user documentation and modified *“SynToxProfiler input”* section, where we described the input file format in more details, especially each of the input file columns (units and formats). Please see <http://syntoxprofiler.fimm.fi/howto/#features>

*- It would also be interesting to compare data analyses pf SynToxProfiler with programs that only do synergy calculations. The benefit would be 2-fold. One, one could compare the synergy values using the same model (Bliss, etc) with 2 software programs (do they agree or not and why not if that’s the case?). Two, the complete STE parameters could then be compared and rated as to the added value of their approach. It looks like they did this within the SynToxProfiler software. One program that might consider to compare to is Combenefit (Bioinformatics, 32(18), 2016, 2866–2868).*

**Our response:** As suggested by the reviewer, we have now compared the synergy scores calculated using SynToxProfiler and Combenefit (new ref 16). SynToxProfiler uses SynergyFinder (ref 21) for synergy assessment*.* We should note that the underlying algorithms for synergy calculations based on a selected synergy model (*e.g. Bliss, Loewe or HSA)* are the same in both SynergyFinder and Combenefit. However, SynergyFinder and Combenefit use different curve-fitting algorithms for the normalization of single drug dose-responses. Additionally, Combenefit normalizes dose-response matrix with an average values of nearest matrix responses, while SynergyFinder uses the baseline (i.e., average of two curves lowest asymptotes) for the full matrix correction. Nevertheless, both algorithms provide similar results with a different scaling of synergy scores. In our comparison, we chose to use Bliss synergy scoring model, which is among the most popular models in drug combination analysis (and since ZIP is not implemented in Combenefit). As expected, we observed a strong correlation between the synergy scores calculated from both tools (Pearson correlation of 0.92 and 0.83, respectively, for the 20 anti-cancer and 77 anti-Ebola drug combinations), indicating that the two software provide similar synergy scores (please see figure below).

We have now added these plots as S3 Fig, and described these results in a new results section (page 5, middle paragraph).

As also suggested by the reviewer, we have now additionally compared top hits ranked by SynToxProfiler combined STE scores with Combenefit’s Bliss synergy scores to demonstrate the added value of using also other components than synergy alone. In the T-PLL data, 7 out of top 10 drug pairs ranked based on Combenefit Bliss synergy score were among the top-10 combinations ranked by SynToxProfiler (see Table below, new S4 Table). Importantly, Buparlisib-Ibrutinib combination was ranked 11^th^ by SynToxProfiler, although it has a higher Combenefit synergy rank (6^th^), because of its lower efficacy and higher toxicity similar to the observation in phase 1 clinical trials for heamatological cancers (in relapsed/refractory diffuse large B-cell lymphoma (DLBCL), mantle cell lymphoma (MCL), and follicular lymphoma (FL), new Ref. 17 and [Clinical trial information: NCT02756247.](http://clinicaltrials.gov/show/NCT02756247)) The lower ranking of less effective and more toxic drug pairs by SynToxProfiler, as compared to Combenefit, suggests that the use of synergy alone for drug pair selection does not de-prioritize toxic and less effective combinations during the top hit selection, and will not allow selection of clinically useful combinations that work by additive mechanisms.

| Combination | STE_SynToxProfiler_ | Rank  (STE_SytnToxProfiler_) | Synergy  _SynToxprofiler_ | Rank  (Synergy_SynToxprofiler_) | Synergy  _Combenefit_ | Rank  (Synergy_combenefit_) |
| --- | --- | --- | --- | --- | --- | --- |
| Cytarabine-Daunorubicin | 0.83 | 1 | 6.33 | 2 | 14.9 | 2 |
| Trametinib-S-63845 | 0.8 | 2 | 4.25 | 1 | 32.0 | 1 |
| Clofarabine-Idarubicin | 0.73 | 3 | 1.32 | 8 | 11.6 | 3 |
| Quizartinib-S-63845 | 0.7 | 4 | -1.05 | 4 | 1.2 | 11 |
| Gefitinib-Trametinib | 0.68 | 5 | -0.44 | 3 | 10.5 | 4 |
| Clofarabine-Prexasertib | 0.68 | 6 | 1.24 | 7 | 7.9 | 5 |
| Ibrutinib-Navitoclax | 0.68 | 7 | 1.61 | 10 | 3.8 | 7 |
| Gefitinib-Omacetaxine | 0.68 | 8 | 0.33 | 11 | 1.5 | 9 |
| Omacetaxine-Ipatasertib | 0.68 | 9 | 0.11 | 12 | 0.3 | 12 |
| Omacetaxine-Alpelisib | 0.65 | 10 | -1.11 | 14 | -6.2 | 17 |
| Buparlisib-Ibrutinib | 0.58 | 11 | -0.44 | 5 | 5.3 | 6 |
| Trametinib-Dasatinib | 0.5 | 12 | -3.73 | 6 | 1.3 | 10 |
| Dasatinib-Ipatasertib | 0.45 | 13 | -0.13 | 9 | 2.2 | 8 |
| Vinorelbine-Clofarabine | 0.4 | 14 | -2.27 | 19 | -19.0 | 18 |
| Clofarabine-Omacetaxine | 0.38 | 15 | -4.85 | 15 | -5.2 | 16 |
| Idarubicin-Ibrutinib | 0.2 | 16 | -1.83 | 13 | -0.1 | 15 |
| Carboplatin-Dexamethasone | 0.16 | 17 | -0.69 | 17 | 0.0 | 14 |
| Ipatasertib-ASP3026 | 0.15 | 18 | -0.02 | 16 | 0.0 | 13 |
| Dexamethasone-Clofarabine | 0.14 | 19 | -18.341 | 18 | -60.5 | 19 |

Similarly, in the anti-Ebola data, SynToxProfiler ranks relatively high (4^th^) colchicine/toremifenecitrate combination, despite its low synergy rank (18^th^) given by Combenefit, because of its higher efficacy. This combination synergistically blocks EBOV infections as colchicine blocks the virus trafficking to endolysosomes and toremifenecitrate blocks its fusion with the cells (Ref. 12), indicating that SynToxProfiler can identify functionally relevant hits that have increased likelihood of success in clinics. This is the added value of SynToxProfiler – that is – to combine synergy and selective efficacy *(efficacy - toxicity)* into ranking of the most promising combinations. We have described these additional comparison results in the new results section (page 5, last paragraph) as S5 table.

We also note that we have compared the overlap in the top hits selected on the basis of synergy and selective efficacy in SynToxProfiler in Figure 2B, which further demonstrates that synergy and selective efficacy are independent drug combination components, which cannot be used alone to prioritize potent and less toxic synergistic drug combinations.

*- Can they provide insights – after using the program on multiple datasets – on drug combinations that show positive synergy, but fail the STE?*

**Our response:** SynToxProfiler ranks lower (i.e., lower STE score) those drug pairs that have lower selective efficacy or synergy, or both. Therefore, synergistic (i.e., showing positive synergy) combinations that have low-efficacy and/or high toxicity will lead to low STE score. For instance, SynToxProfiler ranks low (9^th^) Prexasertib and Clofarabine combinations, despite its higher synergy (4^th^), because of its low efficacy and higher toxicity as observed in Philadelphia-negative B-/T-ALL cell lines where Prexasertib increases the cytotoxicity of clofarabine (new Ref. 13) (page 5, 1^st^ paragraph). Similarly, in the anti-Ebola drug combination screening data, toremifene citrate and apilimod combination is an example of drug pair with lower STE score (0.86 vs 0.96), due to its high toxicity (24.60 vs 13.30), despite having higher synergy (34.16 vs 25.69), in comparison to clomiphene citrate and sertraline HCl combination that was ranked as the top hit (please see Fig.3). These examples are now highlighted (page 5, 1^st^ paragraph).

As drug response and toxicity are both complex traits that involve interaction of multiple pathways in the body, it is very hard to derive a single biological insight that can apply to those drug pairs that failed in STE despite their higher synergy. We believe that each specific case will have a separate mechanism for the low efficacy or high toxicity. Nevertheless, SynToxProfiler can be used to point out such likely successful combinations with low toxicity and high efficacy, regardless of synergy levels, for further study in order to understand the mechanism. To our knowledge, there are no other tools available that allow for profiling of efficacy and potential toxicity of the drug pairs, in addition to their synergy. We have now discussed this benefit in the revised manuscript (page 7, lines 180-182).

***Reviewer #2:*** *The article by Giri and coworkers introduces SynToxProfiler as a tool to help researches investigate drug combinations by taking into account not only synergy/efficacy, but also toxicity. Overall, I like the idea although I have a some minor reservations which I outline below.*

**Our response:** We thank the reviewer for appreciating the added value of SynToxProfiler tool for drug combination screening. Please find below our point-by-point responses to the comments. We have highlighted in yellow all the changes made in the revised manuscript.

1. *An issue with this approach is that the toxicity predictions are only as good as the experimental method used. That is to say that in vitro methods to measure toxicity may not capture clinical toxicity for numerous reasons (eg limited tissue types tested or ADME effects not taken into account). The authors should briefly mention this limitation in the discussion.*

**Our response:** We completely agree that in vitro toxicity measurement may not always capture clinical toxicity, but we argue they are still better than having no toxicity estimation at all. We have now mentioned this as one of the limitations of any toxicity profiling methods in the discussion (page 7, middle paragraph, line 172-180).

*2. Related to the comment above, the introduction would also benefit with a short explanation saying that toxicity is measured on non-diseased cells.*

**Our response:** We have now clarified the introduction (page 2, middle and last paragraphs) and methods section (page 9, first paragraph), explaining that toxicity is measured on non-diseased cells, if available.

*3. It would be helpful to more clearly define (methods section) what the efficacy, toxicity, synergy scores are. Are the efficacy and toxicity scores just the volumes under the dose-response surfaces? What is the synergy score. I have no feeling for what the numbers represent. I recommend including in the introduction a few extra sentences describing how synergy, toxicity and efficacy were calculated and going into significantly more detail in the methods section.*

**Our response:** We have now clarified in the method and introduction sections that the efficacy and toxicity scores are calculated in SynToxProfiler as volumes under the dose-response surfaces measured in diseased and healthy cells, respectively. The synergy score is summarized into single score as a volume under synergy matrix, calculated using appropriate synergy model (e.g. Bliss, Loewe, or HSA) from the dose-response surfaces measured in diseased cells. The efficacy, toxicity and synergy scores represent the summarized efficacy, toxicity and synergy level for a drug pair combined at multiple concentration (e.g.8x8 matrix design), and measured in a particular patient/cell line. The higher score represents higher efficacy, toxicity or synergy of a drug pair. We have now clarified this under introduction (page 2, middle and last paragraphs) and method sections (page 9, 1^st^ paragraph).

To better illustrate the step-by-step scoring procedure with real input data, we have also added the below schematic illustration as S4 Fig and into the SynToxProfiler User Documentation (<https://syntoxprofiler.fimm.fi/howto>). In this example, the expected matrix is calculated using Bliss synergy model, but the tool implements also the other synergy scoring models available for the user to choose depending on the drugs and hypotheses of the study (page 9, 1^st^ paragraph)

*4. Is volume under the dose response curve an appropriate metric for measuring efficacy of a combination - does this ignore the shape of the dose response surface? For example I can imagine a situation in which when drugs are combined at a certain ratio they are particularly toxic, but at a different ratio they are not toxic. The latter ratio might be therapeutically useful, but this could be missed if only volume is considered because this would be an “average” of the toxic and non-toxic ratios. Please consider whether it is appropriate to include this in the discussion – possibly with a recommendation to users to look at individual response surfaces and not just the overall score when selecting drug combinations to investigate?*

**Our response:** The approach for measuring efficacy of drug combination as volume under the dose-response surface has been adopted from the popular concept of area under the dose-response curve *(AUC),* when efficacy of a single drug is measured at multiple concentrations. The integrated volume under dose-response surface captures the variation in the response at different doses for different drug combinations in screening, and it also effectively normalizes for the dose-range differences in order to adjust the potential bias in dose selection. It can also capture the shape of the curves. For example, drug pair that have response ranges covering the asymptotic limit will have higher score as compared to pairs that have less response. However, we are open to implement any alternative approach to measure efficacy of drug pairs in the future versions of the tool.

We agree with the reviewer that summarization of efficacy/toxicity level measured at different concentrations into a single volume score may not capture the full response changes at specific concentrations. However, the main aim of SynToxProfiler is to summarize the dose-response matrix data and prioritize top drug combinations with overall high synergy and efficacy but low toxicity. In order to rank multiple drug combinations measured at different dose-ranges in a high-throughput screening, summarization of the measurements across different concentrations in to a single score appears as an appropriate approach. As suggested by the reviewer, we have added into the revised discussion a recommendation to users to look at full response surfaces, and not just the overall score, when selecting drug combinations to investigate (page 7, 1^st^ paragraph).

Further, SynToxProfiler calculates the STE scores not only for the whole matrix (by taking volumes under dose response surface), but also for the most synergistic concentration window (volumes are taken only at 3x3 most synergistic concentration range of each drug combination, default option in SynToxProfiler). This allows for the comparison of drugs at their most beneficial concentration ranges for those combination screens where the primary aim is to find most synergistic drug pairs. Moreover, in the analysis output, we provide individual matrices for efficacy, toxicity and synergy estimates at all measured doses (highlighting the 3x3 most synergistic concentration range), so that users can confirm efficacy/synergy/toxicity at individual dose for selection of top hits for further study. We have included also this recommendation in discussion (page 7, 1^st^ paragraph).

*5. I think that the method of validating the SynToxProfiler is a little weak – in essence by showing that it predicts a few successful clinical combinations. However, I can think of no better way to validate it. Perhaps the authors could add a sentence in the discussion saying that the validity of the tool will hopefully be demonstrated as other scientists start to use it?*

**Our response:** We totally agree with the reviewer. Even though the manuscript lacks a very systematic validation of the method, we feel that successful identification of clinically established drug combination (e.g. Daunirubicin-cytarabine in T-PLL screening and clomifene-sertraline in anti-Ebola screening) is the most important outcome of the tool. We have added a sentence stating that the “The validity of the tool will hopefully be demonstrated as other scientists start using SynToxProfiler.” (page 7, last paragraph).

*6. The figure numbering and order is mixed up.*

**Our response**: Thank you for pointing this out. We have corrected it in the revised version.

*7. Define ZIP abbreviation*

**Our response**: We have now defined the ZIP abbreviation (page 2, middle paragraph).

*8. Define colour coding fig 1A*

**Our response:** We have added a color coding for Fig 1A (now Fig. 2A) and described it in the legend.

*9. Fig 1 seems to suggest a good toxicity score is good – I am struggling with this if it’s the volume under the toxicity curve.*

**Our response:** We thank the reviewer for pointing this out. We have now clarified the legend of the Fig 1 (now Fig. 2) for better understanding of the colors used. The vertical axis (Z-axis) in the figure represents the STE score which is not volume under toxicity curve. STE is a score integrating efficacy, synergy and toxicity of a drug combinations that has been used in SynToxProfiler for ranking the combination. Higher STE score represents higher efficacy, higher synergy and lower toxicity of a drug pair. We have now clarified it in the method section (page 9, 1^st^ paragraph)

Additionally, in the new Fig 1 (old Fig 3). we have reversed the toxicity scale so that lower *toxicity* corresponds to higher *STE score*.
